# The failure to increase social support: it just might be time to stop intervening (and start rigorously observing)

**DOI:** 10.1007/s13142-016-0458-9

**Published:** 2017-01-09

**Authors:** Amanda L. Graham, George D. Papandonatos, Kang Zhao

**Affiliations:** 1 Schroeder Institute for Tobacco Research and Policy Studies, Truth Initiative, 900 G St NW, Fourth Floor, Washington, DC 20001 USA; 2 Department of Oncology, Georgetown University Medical Center/Cancer Prevention and Control Program, Lombardi Comprehensive Cancer Center, Washington, DC USA; 3 Center for Statistical Sciences, Brown University, Providence, RI USA; 4 Tippie College of Business, The University of Iowa, Iowa City, IA USA

**Keywords:** Social network, Social support, Smoking cessation, Internet

## Abstract

In 1986, Lichtenstein et al. (*Behav Ther*. 17(5):607–19, 1986) presented the results of five studies focused on enhancing social support for smoking cessation in community-based clinic and worksite interventions. The manuscript was titled Social Support in Smoking Cessation: In Search of Effective Interventions and its main conclusion was that "attempts to both increase social support and to enhance treatment effectiveness have not been successful." Thirty years later, the paper by Cutrona et al. (*Transl Behav Med*. 6(4):546–57, 2016) draws a similar conclusion from a study focused on providing social support through an online social network for smoking cessation. In reviewing these findings - and based on our knowledge of the extensive literature on social support interventions that has been published over the past 30+ years - we believe there is a need for a fundamental shift in research on social support. Our focus here is largely on smoking cessation, but our comments are applicable to other areas of behavior change.

## INVITED COMMENTARY

In 1986, Lichtenstein et al. [1] presented the results of five studies focused on enhancing social support for smoking cessation in community-based clinic and worksite interventions. The manuscript was titled *Social Support in Smoking Cessation: In Search of Effective Interventions*, and its main conclusion was that “attempts to both increase social support and to enhance treatment effectiveness have not been successful.” Thirty years later, the paper by Cutrona et al. [2] draws a similar conclusion from a study focused on providing social support through an online social network for smoking cessation. In reviewing these findings—and based on our knowledge of the extensive literature on social support interventions that has been published over the past 30+ years—we believe there is a need for a fundamental shift in research on social support. Our focus here is largely on smoking cessation, but our comments are applicable to other areas of behavior change.

In the years since the study of Lichtenstein et al. [1], dozens of studies designed to create, augment, or otherwise manipulate social support in the interest of improving quit rates have yielded disappointing results [3, 4]. Group-based smoking cessation interventions typically assemble smokers on the basis of a shared quit date along with geographic and chronological availability. The notion behind group interventions is that members will create meaningful social relationships and that the exchange of information and support will drive behavior change. A systematic review of 55 trials [5] found no evidence that social interactions were a critical element in the success of group interventions or that manipulating social support in group interventions had an effect on smoking outcomes. Buddy interventions pair smokers with an individual who has special responsibility to support the smoker in their efforts to quit. The buddy may be a non-smoker, another smoker trying to stop, an ex-smoker, or even a current smoker. Most trials have involved individuals from an existing social structure (i.e., friend, living partner, spouse), though some studies have evaluated the impact of creating a new social tie during the study period. Several systematic reviews have found that buddy/partner/spouse interventions failed to consistently increase quit rates via increases in perceived support measures [6–8].

With the widespread proliferation of social media and online social networks, there has been renewed interest in evaluating the role of social support in behavior change through online interventions. Conceptually, online social networks have the potential to overcome many of the challenges of social support interventions conducted in face-to-face settings [9]. The relative anonymity of the Internet may facilitate the exchange of support in online communications. Online communities can be a “safe space” for smokers to talk about the challenges of quitting and the day-to-day stressors that trigger cravings and relapse. For many smokers, offline social relationships involve cues to smoke and stressful interpersonal interactions; online interactions may be the only place where they receive consistent and positive encouragement around quitting. Additionally, in large-scale established social networks, there are many individuals available at a given time to share information, commiserate, cheer, coach, celebrate, etc. It may be that a single support person can provide some of these support functions but cannot possibly meet all of the varying needs of someone making a major health change. Online networks tend to be comprised of individuals who can provide support at varying points along the continuum of behavior change or illness management. In the case of smoking cessation, former smokers can model abstinence behaviors and share their insights about how to quit, while current smokers can empathize and provide a common shared experience around quitting. Perhaps most importantly, online social networks are unlike any other treatment modality in that they are available more persistently, across geographic boundaries, and can provide immediate support precisely at the time that users are looking for it, often for as long as they desire.

The study by Cutrona et al. [2] attempted to leverage the availability of an existing, large online social network for smoking cessation to improve quit rates. Participants enrolled in the Decide2Quit.org smoking cessation website were offered the opportunity to visit and join the online community that is part of the BecomeAnEX.org smoking cessation website. The authors found that only one in four Decide2Quit users clicked through to BecomeAnEX, only 7.5% registered, and the level of engagement and exposure on BecomeAnEX was too low to create a clinically meaningful impact on abstinence. Considering these findings alongside other studies of online social networks—and the wealth of research that has been done in “offline” settings—we have identified three “lessons learned” that are relevant for the study of online social networks. Given that research on online networks is very much still in its infancy, investigators would be wise to benefit from existing knowledge on social support, social networks, and community building.

1. “Community is the noun, online is the fancy dressing.” In his *Online Community Manifesto*, Millington [10] calls for a shift in the development and management of online communities away from a fascination with the “fancy dressing” of technology and toward a more sophisticated understanding of community users. What motivates people to visit an online community in the first place? What makes them want to contribute? What role do intrinsic and extrinsic motivation play in visiting an online social network and subsequent engagement? How do groups form and interact? What makes people stick around long after they achieved their initial goals? How do online communities evolve from a collection of haphazard posts to a recognizable network with defined user roles and meaningful patterns of engagement? An unfortunate number of Internet-based research studies have side-stepped these kinds of questions and moved directly to testing various types of social support interventions, many of which may be neither social nor supportive. It is critical to have a deep understanding of the complex intrapersonal, interpersonal, and sociological elements at play in community development before focusing on their application via technology.

On its face, the approach that Cutrona et al. [2] took in linking to an existing online social network makes good sense. It can take years to develop and nurture an online cessation social network until it reaches a point of maturity with a critical mass of active members [11]. The vast majority of online communities never reach this point, attracting only a handful of posts per month in what users then experience as an “empty room” [11, 12] and an “inert” intervention.

The authors note that the need to register on Decide2Quit and again on BecomeAnEX may have acted as a deterrent to full participation in the online community. We agree that this was a limitation of the study, and offer an additional perspective. Successful online communities are dynamic, living entities that facilitate participation because users find value in others’ contributions, feel a sense of connection and shared experience with other members, and feel safe in participating. This is true for any community, online or offline. For online communities, in particular, the technology needs to sit quietly in the background, seamlessly facilitating these social processes. The low click-through rates and even lower engagement rates reported in Cutrona et al. [2] may have little to do with the online social network itself and more to do with the implementation approach. Qualitative research has a critical role to play in understanding the user experience related to the implementation of social support interventions and evaluation of their effectiveness.

2. “Nature knows best.” While not a scientific construct per se, the importance of interpersonal “chemistry” cannot be overlooked in this line of research. We choose who to befriend among our neighbors, our coworkers, and the people we encounter through shared experiences like parenting, sports, book clubs, and quilting. Whether or not someone seeks out interpersonal support or social contact as they make lifestyle changes is influenced by a whole host of factors, many of which may not be malleable or amenable to intervention. These factors may include past experiences, personality characteristics, complex psychological constructs like outcome expectations and trust, social competence or interactional skills, the nature of the health behavior itself, and potential stigma that may surround it, to name just a few. There is even emerging evidence of an association between the structural and functional properties of the human brain and the size and complexity of social networks [13]. For some people, enlisting existing connections (e.g., friends, family, coworkers) for support during health behavior change may be infeasible, complicated, or risky. For others, harnessing the support of complete strangers through an online social network is a completely foreign concept. And for a sizable proportion of people, involving anyone at all in their efforts at health behavior change may be counterintuitive, awkward, or feel forced. There are some people who simply choose to “go it alone.”

Decades of research on social support interventions may simply be telling us that social support is not for everyone, and among those for whom it is important, the social relationships that form naturally yield the greatest benefit. Social interactions should not be presumed synonymous with social relationships nor should they be expected to have the same impact on perceived support, received support, and health behavior change. Furthermore, interactions with “grafted” helpers may be fundamentally different than interactions with “indigenous” helpers [14]. These are not new revelations. In 1991, Barerra extended Commoner’s [15] Third Law of Ecology (“nature knows best”) to the study of social support interventions, noting that “interventions that attempt to mimic naturally occurring processes can miss elements that are essential for their effectiveness” (p. 134, [16]). We surface these ideas again as they relate to online interventions to caution that social relationships cannot be “assigned” regardless of the methodological rigor of any randomized controlled trial. Researchers and practitioners alike should remain open to the idea that developing or augmenting other kinds of cognitive and behavioral coping skills may be more fruitful than attempting to manipulate the social environment, especially for individuals that are not interested in or not naturally drawn to relying on social relationships during health behavior change.

3. “Well-designed observational methods offer an alternative to doing nothing.” As noted by Black [17], observational research methods have much to contribute to the study of many aspects of healthcare. While he explicitly referred to “quantitative, epidemiological methods” in his 1996 commentary, observational methods today include a range of new statistical approaches and modern computational social science methods that open new doors for the study of online social networks.

As mentioned by Cutrona et al. [2], analysis of randomized trials of smoking cessation treatments has moved away from the traditional “missing equals smoking” assumption and now routinely deals with threats to randomization posed by study dropout with sophisticated statistical methods such as inverse probability of dropout weighting (IPDW) [18]. However, in observational studies aimed at evaluating the effectiveness of various social support strategies, social support utilization levels are not assigned at random but are chosen freely by study participants. In order to make causal statements regarding the impact of changes in utilization levels, it is imperative that we first observe what people do of their own volition, and then apply appropriate statistical methods to evaluate the impact of those actions. For example, in the study of Cutrona et al., participants self-selected whether to visit BecomeAnEX at all and then further self-selected their level of engagement (i.e., register, browse, actively participate in the community). Therefore, reported differences in abstinence rates lack a causal interpretation, as they fail to account for treatment selection bias. Additional inverse probability of treatment weighting (IPTW) is needed to further correct observed abstinence rates for participants’ differential propensity to participate in the community, essentially recreating a randomized experiment [19, 20]. One can combine the IPDW with IPTW approaches by jointly modeling the dropout and treatment selection process using either nested logistic regression modeling (marginal probability of dropout × conditional probability of community use) or multinomial logistic regression, with dropout × community use combinations modeled as a multicategory nominal outcome. The latter is more flexible as it does not impose any temporal precedence assumptions. Estimating these models non-parametrically using generalized boosted models (GBMs) as implemented in the GBM package [21] adds robustness to possible propensity model misspecification, compared to the use of parametric logistic regression modeling.

Further, it appears that Cutrona et al. [2] did not check whether their reweighing scheme was effective in achieving balance in baseline covariates across study dropouts and completers. Propensity weights should be inspected routinely to assess the covariate overlap and the need for weight trimming [22], aided by the balance assessment diagnostics of packages such as TWANG [23]. If absolute standardized bias measures [24] remain elevated, one should seek to improve the propensity model in an iterative fashion and/or note its failure to achieve the desired level of bias correction.

Finally, outcome modeling should correctly treat the propensity weights as probability weights and not precision weights (e.g., via the use of survey packages [25]). Otherwise, standard errors will be estimated incorrectly, leading to misleading conclusions as to the statistical significance of the study findings. One strong caveat remains: unlike randomized trials, propensity score adjustment of observational studies cannot achieve balance over unmeasured confounders uncorrelated with variables included in the propensity model specification. Therefore, researchers should do their utmost at the study design phase to measure not only potential confounders of the treatment-outcome relationship but also strong predictors of study dropout and treatment utilization. It is also worth noting the opportunity for the development of new measurement instruments designed specifically for the online environment, rather than simply adapting measures originally developed to assess offline social support.

Research also needs to focus on the mechanisms through which greater engagement in online social networks is theorized to impact behavior change rather than simply the overall effect of engagement itself. A 2011 manuscript by Thoits titled *Mechanisms Linking Social Ties and Support to Physical and Mental Health* [26] is a must-read in this regard. Attending to mechanisms will require careful consideration of measures, many of which are articulated by Thoits but remain largely underutilized. Answering questions about “For whom?” and “Under what conditions?” is critical to this work, as is defining what effective engagement means for online social networks [27]. It is possible to address these types of questions by applying computational social science methods to large-scale datasets from online social networks. Online social networks track when and with whom interactions occur for a large number of individuals during an extended period of time. These data allow us to conduct dynamic social network analyses to better understand how natural interpersonal relationships form and strengthen, discern patterns among people that choose to affiliate, when in the course of the social network engagement that affiliation occurs, when those ties dissolve, and individuals’ trajectories in their social network engagement [28]. The content of individuals’ interactions is also available in online social networks, providing exciting opportunities for using text mining to understand what people talk about or share in cyberspace. Techniques such as text classification, topic models, and sentiment analyses can enrich social network analyses by revealing whether the formation of social ties is related to engagement around specific topics, whether different types of social support (e.g., informational vs. emotional support) are differentially related to long-term engagement, whether similarities or differences in opinions on a particular topic or changes in views are predictive of the formation or dissolution of social ties, and evidence of social influence on health-related opinions or behaviors [29–31]. The integration of social network analyses and text mining to analyze data from an online social network also has the potential to better predict individuals’ future engagement in the social network and their offline behaviors. These are fundamentally different questions than evaluating the effectiveness of a social support intervention, but they are ones that we believe have much greater potential to advance our understanding of the ways that social ties can influence behavior.

Recognizing that social interventions are not for everyone is not to suggest that they are for no one. Hundreds of thousands of current and former smokers connect each year in online communities offered through state quitlines [32], employers and health plans [33], and freely available programs in the USA and abroad [34–36], as well as on social networking sites such as Facebook [37] and Reddit [38]. Online communities also provide information and support to the countless numbers of people who turn to the Internet for assistance with addictions [39], weight loss and nutrition [40], chronic disease [41], and many other health conditions. In 2007, Hesse and Schneiderman [42] noted that “The field of computer science is littered with applications that, although technologically superior, have failed to find a following among discriminating users.” We would argue that today, the Internet is littered with online social networks that, though widely used and valued by users, have failed to find a following among researchers. Given the range of tools and rigorous methodological approaches at our disposal, there is an exciting opportunity to study social relationships through a new lens.
